# Supplementary material for: The erosion of large primary atmospheres typically leaves behind substantial secondary atmospheres on temperate rocky planets
Source: Nat Commun. 2024 Sep 27;15:8374. doi: 10.1038/s41467-024-52642-6 (PMC11437211; doi:10.1038/s41467-024-52642-6)
Supplement: Supplementary file 1 — Supplementary Information [file 41467_2024_52642_MOESM1_ESM.pdf]

Supplementary Materials

The erosion of large primary atmospheres typically leaves behind substantial secondary atmospheres on temperate rocky planets

Joshua Krissansen-Totton[1,2]*, Nicholas Wogan[2,3], Maggie Thompson[4], Jonathan J. Fortney[5]
[1]Department of Earth and Space Sciences/Astrobiology Program, University of Washington, Seattle WA, USA

[2]NASA NExSS Virtual Planetary Laboratory, University of Washington, Seattle, WA 98195, USA

[3]NASA Ames Research Center, Moffett Field, CA, USA

[4]Department of Earth Sciences, ETH Zurich, Switzerland

[5]Department of Astronomy and Astrophysics, University of California, Santa Cruz, Santa Cruz, CA 95064, USA

*Contact: joshkt@uw.edu

Model Validation

Earth and Venus Validation

Fig. S1 and S2 show the magma ocean model applied to an Earth and Venus magma ocean, respectively. Here, we assume the traditional view whereby Earth and Venus do not form with a substantial H_2 envelope and are left with only a few Earth oceans equivalent of H following the moon-forming impact, in the case of the Earth (and similar volatile endowments for Venus). Consistent with recent studies of Earth's magma ocean, it takes around ~1 Myr for the magma ocean to solidify, and most water is retained in the mantle due to melt trapping and the high solubility of water in silicate melts [1-4]. The atmosphere that emerges from the magma ocean is CO_2-dominated, with some CO and a small amount of liquid water (5-10% of an Earth ocean) on the surface. Note that in this calculation Earth's surface water is all lost to space since we do not consider subsequent degassing of water in the mantle [1], nor include a temperate carbon cycle to draw down CO_2 [5, 6]. The validation test for Venus is similar except that, for our assumed Bond albedo, water does not condense due to higher insolation, leading to the eventual desiccation by the loss of water to space. We leave a more detailed Earth-Venus comparison with this model for future work, but note that this generalization of magma ocean models to all C, H, O, and Fe-bearing species does not dramatically change the classical view of the Earth-Venus evolutionary dichotomy [7] when relatively oxidized starting conditions and chondritic compositions are assumed.

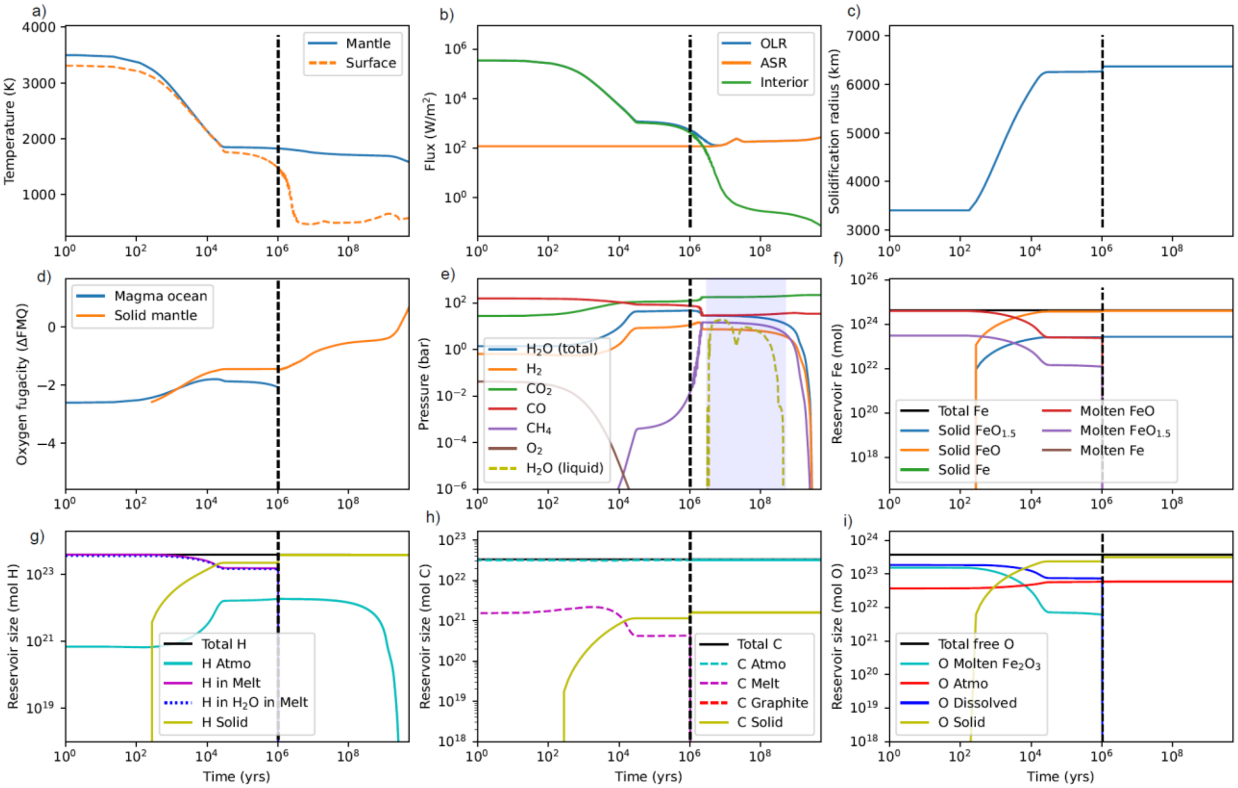

Fig. S1: Nominal Earth. Subplot (a) denotes the time-evolution of surface (orange) and mantle potential temperature (blue), (b) denotes the evolution of outgoing longwave radiation (OLR, blue), absorbed shortwave radiation (ASR, orange), and interior heatflow (green), and subplot (c) shows the evolution of the magma ocean solidification front from the core-mantle boundary to the surface. Subplot (d) shows solid mantle (orange) and magma ocean redox (blue) relative to the Fayalite-Quartz-Magnetite (FMQ)

buffer, (e) shows the evolution of atmospheric composition including H_2O, H_2, CO_2, CO, CH_4, O_2, and condensed/liquid H_2O. Subplot (f) denotes iron speciation in both the magma ocean and the solid silicate mantle – in this oxidizing Earth-analog case no metallic iron is produced. Subplot (g) shows both solid and fluid reservoirs of H; total dissolved hydrogen (purple) and hydrogen dissolved as H_2O (blue-dotted) are essentially identical in this oxidized scenario where dissolved H_2 is minimal. Subplot (h) denotes solid and fluid reservoirs of C and subplot (i) denotes solid and fluid reservoirs of free oxygen, including oxygen bound to ferric iron, atmospheric species, and O in volatiles dissolved in the melt (H_2O, CO_2) reservoirs respectively. Vertical dashed black lines show the termination of the magma ocean, which takes ~10^6 years in this case. The blue shaded region denotes the timespan over which liquid water is stable on the surface – this provides a window of opportunity for H_2O degassing via volcanism, and the removal of CO_2 via weathering to form carbonates (neither of which are included in this model).

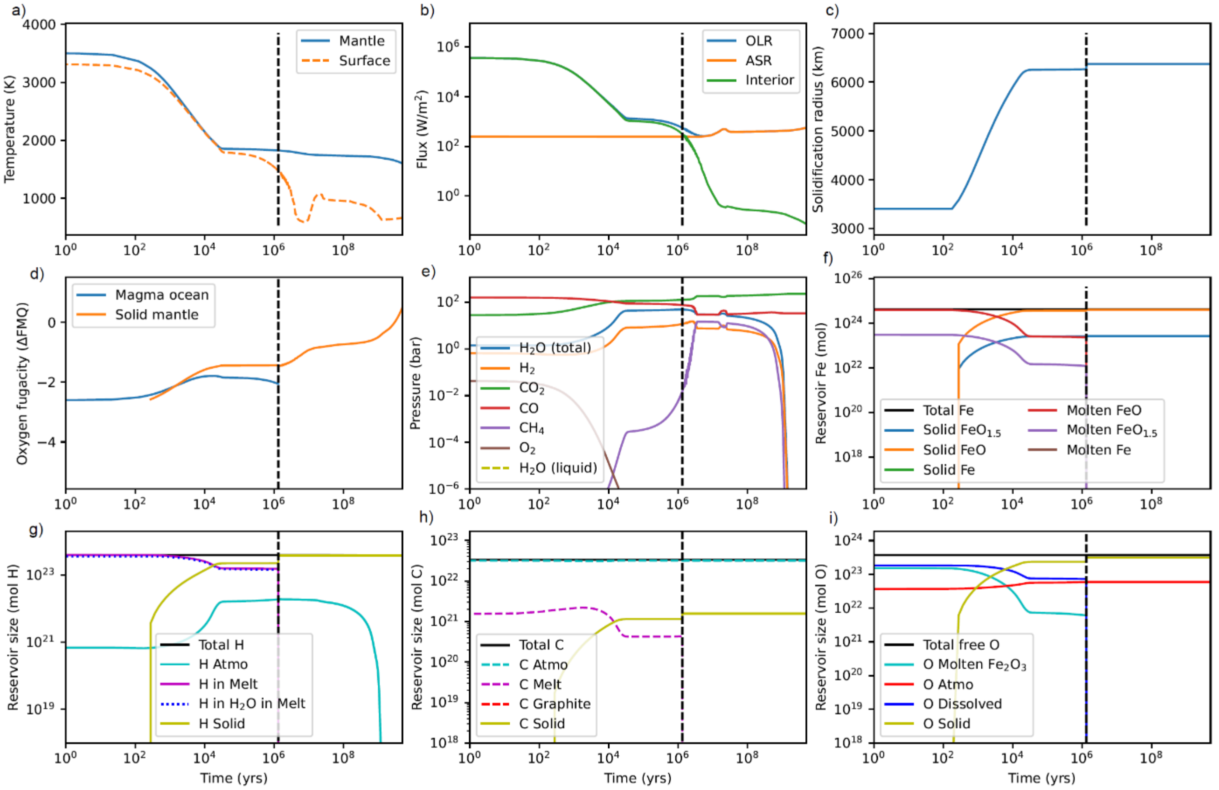

Fig. S2: Nominal Venus. Subplot (a) denotes the time-evolution of surface (orange) and mantle potential temperature (blue), (b) denotes the evolution of outgoing longwave radiation (OLR, blue), absorbed shortwave radiation (ASR, orange), and interior heatflow (green), and subplot (c) shows the evolution of the magma ocean solidification front from the core-mantle boundary to the surface. Subplot (d) shows solid mantle (orange) and magma ocean redox (blue) relative to the Fayalite-Quartz-Magnetite (FMQ) buffer, (e) shows the evolution of atmospheric composition including H_2O, H_2, CO_2, CO, CH_4, O_2, atmospheric (non-condensed) H_2O. Subplot (f) denotes iron speciation in both the magma ocean and the solid silicate mantle – in this oxidizing Earth-analog case no metallic iron is produced. Subplot (g) shows both solid and fluid reservoirs of H; total dissolved hydrogen (purple) and hydrogen dissolved as H_2O (blue-dotted) are essentially identical in this oxidized scenario where dissolved H_2 is minimal. Subplot (h) denotes solid and fluid reservoirs of C and subplot (i) denotes solid and fluid reservoirs of free oxygen, including oxygen bound to ferric iron, atmospheric species, and O in volatiles dissolved in the melt (H_2O, CO_2) reservoirs respectively. Vertical dashed black lines show the termination of the

magma ocean, which takes ~10^6 years in this case. Liquid water is not stable on the surface at any point in this particular model run, and so hydrogen is lost to space until Venus is completely desiccated.

Note that for both the Earth-analog and Venus-analog calculations above around ~2-10% of CO_2 is retained in the mantle, broadly in line with previous studies [8, 9], as well as the TRAPPIST-1e results with BSE volatile endowments (Fig. 2). Far less C is retained in the mantle in Fig. 3 and 4, which is attributable to the reducing conditions created by the nebular atmosphere. Under reducing conditions, CO_2 is a small fraction of the total atmospheric carbon, and so the resultant dissolved carbonate melt fraction is similarly low. We neglect CH_4 and CO solubility – a conservative assumption that prevents reduced forms of C dissolving in the magma ocean, potentially shielding it from escape.

Volatile-Partitioning Validation

Fig. S3 shows an example volatile partitioning + redox equilibration calculation for a test case with 10^{22} kg free oxygen, 2×10^{24} kg melt, 1500°C, and 100 ppm C (initial melt fraction). The initial H_2 melt fraction was varied from 10 ppm to 1%, and the multiphase equilibrium state is calculated at each point as described in Methods. Subplots denote gas phase partial pressures, dissolved species concentrations, iron speciation in the melt, and melt oxygen fugacity relative to the Iron-Wustite buffer, respectively. Conditions are relatively oxidizing at low initial H i.e. CO_2 dominates the atmosphere, ~half of iron oxidized to $FeO_{1.5}$, and an oxygen fugacity around IW+7.5. However, as the initial H inventory is increased the atmosphere transitions to CO-dominated, then H_2 dominated. Iron speciation transitions from an $FeO_{1.5}$-FeO system to a FeO-Fe system, and melt oxygen fugacity approaches IW-3.

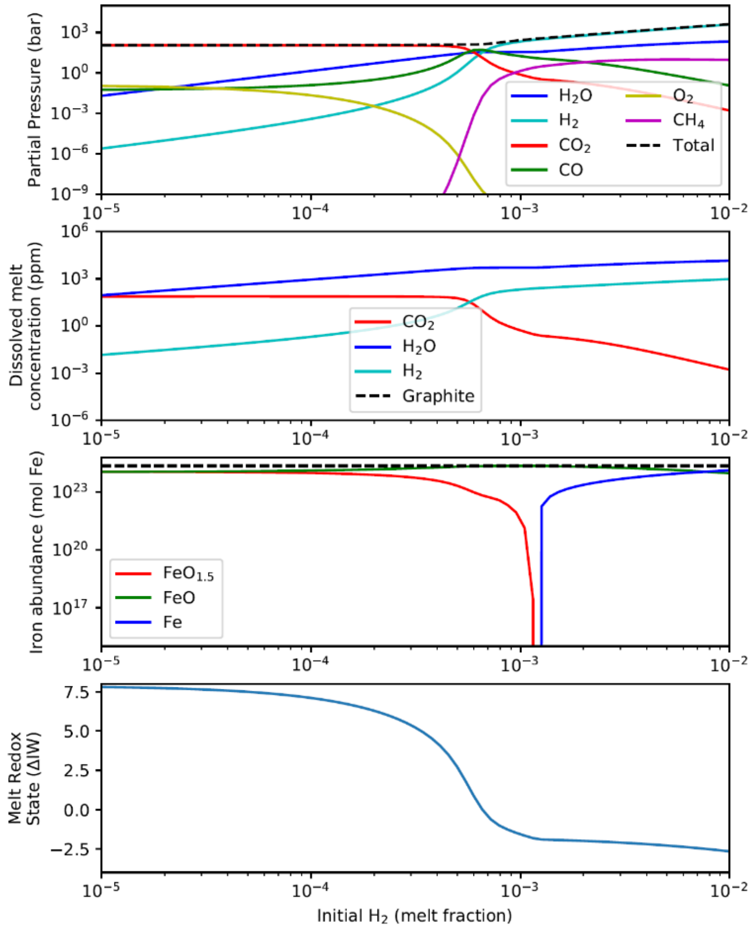

Fig. S3: Example multiphase equilibrium and volatile partitioning calculation. In this case the planet is endowed with 10^{22} kg free oxygen, 2×10^{24} kg silicate melt (half Earth's mantle), 100 ppm C, and calculations are performed at 1500°C. From top to bottom, subplots denote atmospheric species, volatiles dissolved in the silicate melt, iron speciation in the melt, and melt redox state relative to IW.

Fig. S4 is similar except that the initial C endowment is higher (1000 ppm), and there is less free oxygen 5×10^{21} kg). Under these more reducing, carbon-rich conditions, a CO-rich atmosphere is expected for low initial H, and graphite can precipitate out of the melt. Note, however, that under extremely reducing conditions CH_4 dominates over CO and CO_2 and the melt is no longer graphite saturated.

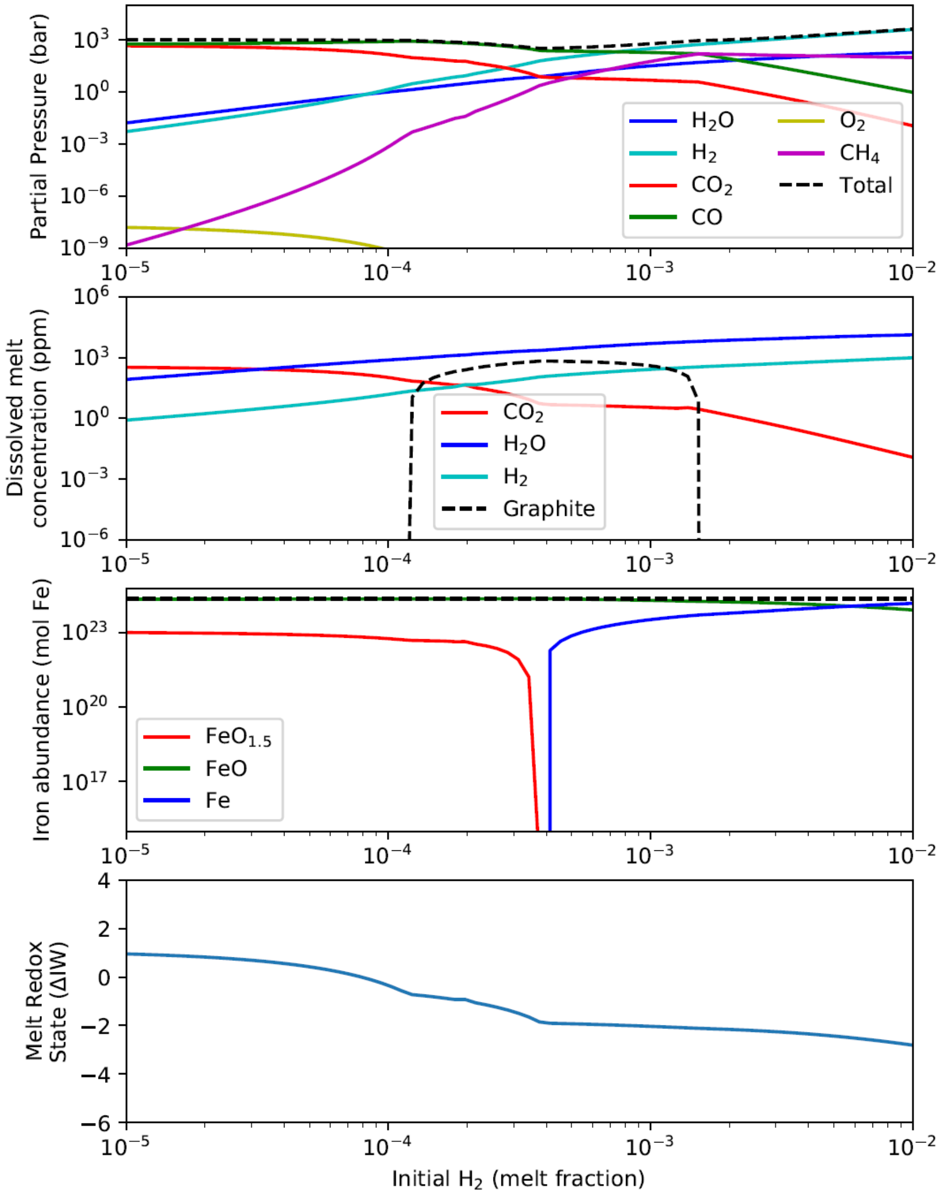

Fig. S4: Example multiphase equilibrium and volatile partitioning calculation. In this case the planet is endowed with 5×10^{21} kg free oxygen, 2×10^{24} kg silicate melt (half Earth's mantle), and 1000 ppm C, and calculations are performed at 1500°C. From top to bottom, subplots denote atmospheric species, volatiles dissolved in the silicate melt, iron speciation in the melt, and melt redox state relative to IW.

Supplementary Methods

Graphite saturation

Following Ortenzi et al. [10], we permit graphite saturation when calculating melt-solid-gaseous partitioning of carbon. The concentration of carbon dissolved in a graphite-saturated melt in redox state dependent:

$$m_{CO_3,graphite-sat} = \frac{K_{1,graphite} K_{2,graphite}\, fO_2}{1 + K_{1,graphite} K_{2,graphite}\, fO_2} \quad (1)$$

$$m_{CO_2,graphite-sat} = \frac{(44/36.594)\, m_{CO_3,graphite-sat}}{1-(1-44/36.594) m_{CO_3,graphite-sat}} \quad (2)$$

Here, fO_2 is mantle oxygen fugacity, and we are converting between dissolved carbonate and carbon dioxide concentrations. The temperature and pressure-dependent equilibrium constants are defined as follows:

$$\begin{aligned} log_{10}(K_{1,graphite}) &= 40.07639 - 2.53932\times10^{-2}\,\mathrm{T} + 5.27096\times10^{-6}\,\mathrm{T}^2 + 0.0267(\mathrm{P}-1\ )/\mathrm{T} \\ log_{10}(K_{2,graphite}) &= -6.24763 - 282.56/\mathrm{T} - 0.119242(\mathrm{P}-1000)/\mathrm{T} \end{aligned} \quad (3)$$

When computing melt-volatile-redox equilibrium, if $m_{CO_2, graphite-sat} > m_{CO_2}$ then the melt is assumed undersaturated with respect to graphite and $n_{graphite}$=0. Otherwise, dissolved CO_2 concentration is adjusted downwards such that $m_{CO_2} = m_{CO_2, graphite-sat}$, and $n_{graphite}$ is included in the carbon mass balance.

Transition between iron speciation parameterizations

Recall that for comparatively oxidized silicates, the relationship between ferrous and ferric iron abundances is determined by oxygen fugacity [11]:

$$\ln\left(\frac{X_{FeO_{1.5}}}{X_{FeO}}\right) = 0.196\times\ln(fO_2) - 1.828 X_{FeO_{Tot}} + {}^{11492}\!/_{T} - 6.675 - 2.243 X_{Al_2O_3} + 3.201 X_{CaO}$$
$$+5.854 X_{Na_2O} + 6.215 X_{K_2O} - 3.36\left(1 - \frac{1673}{T} - \ln\left(\frac{T}{1673}\right)\right) - 7.01\times10^{-7}\frac{P}{T} - 1.54\times10^{-10} P \frac{(T-1673)}{T}$$
$$+3.85\times10^{-17}\frac{P^2}{T}$$

(4)

Here, X_i represent the mole fraction of the *i*-th species in the silicate melt where non Fe-bearing species are fixed bulk silicate Earth values [12], *P* (Pa) is pressure, and T (K) is the melt temperature. We assume metallic iron is negligible for this oxidized case.

For reducing melts, ferrous iron and metallic iron abundances are related to oxygen fugacity as follows:

$$2\times\log_{10}\left(\frac{1.5X_{FeO}}{0.8X_{Fe}}\right)=\log_{10}\left(fO_2\right)-\log_{10}\left(fO_2\left(IW\right)\right) \tag{5}$$

Here, ferric iron is assumed negligible, the mole fraction of iron in the metallic phase, X_{Fe} equals 1, and activity coefficients representative of typical surface conditions have been chosen for iron oxide, 1.5, and metallic iron, 0.8 [13, 14]. The oxygen fugacity at the iron wustite (IW) buffer is calculated from surface temperature and pressure using [15]:

$$\log_{10}\left(fO_2\left(IW\right)\right)=\frac{-27215}{T}+6.57+0.0552\frac{\left(P-1\right)}{T} \tag{6}$$

According to this equation, metallic iron begins to form in the melt when oxygen fugacity is less than:

$$fO_2\left(transition\right)=10^{\left(2\log_{10}\left(\frac{1.5XFeO_{Tot}}{0.8}\right)+\log_{10}\left(fO_2\left(IW\right)\right)\right)} \tag{7}$$

For oxygen fugacities exceeding 10x this transition oxygen fugacity, we assume iron speciation is governed entirely by equation (4). For, oxygen fugacities less than the transition oxygen fugacity, $XFeO_{1.5}$ = 0 and iron is partitioned between metallic and ferrous phases by equation (5). For intermediate oxygen fugacities, metallic iron is zero but we make the following smooth interpolation for numerical efficiency:

$$\ln\left(\frac{X_{FeO_{1.5}}}{X_{FeO}}\right)=0.196\times\ln\left(fO_2\right)-1.828X_{FeO_{Tot}}+11492/T-6.675-2.243X_{Al_2O_3}+3.201X_{CaO}+5.854X_{Na_2O}$$
$$+6.215X_{K_2O}-3.36\left(1-\frac{1673}{T}-\ln\left(\frac{T}{1673}\right)\right)-7.01\times10^{-7}\frac{P}{T}-1.54\times10^{-10}P\frac{\left(T-1673\right)}{T}+3.85\times10^{-17}\frac{P^2}{T}$$
$$+1-\left(\frac{1}{\log_{10}\left(fO_2\right)-\log_{10}\left(fO_2\left(transition\right)\right)}\right)$$

(8)

The final term ensures that $XFeO_{1.5}$ smoothly goes to zero as the iron wustite buffer is approached, and tends to zero as oxygen fugacity approaches ten times the transition abundance.

Escape parameterization

Atmospheric thermal escape is assumed to be either diffusion-limited or XUV-limited, depending on atmospheric composition and the incident stellar XUV flux. In the diffusion limit, we assume that eddy diffusion dominates vertical transport at altitudes where water is more abundant than atomic H and O [16], and so escape of hydrogen is limited by the diffusion of atomic hydrogen through the background atmosphere:

$$\varphi_{diff}=b_H f_H\left(\frac{1}{H_n}-\frac{1}{H_H}\right) \tag{9}$$

$$b_H = \frac{b_{H-CO}\,pCO + b_{H-N_2}\,pN_2 + b_{H-O}\,pO}{pCO + pN_2 + pO} \tag{10}$$

$$H_H = \frac{8.314 T_{cold-trap}}{M_H g} \tag{11}$$

$$H_n = \frac{8.314 T_{cold-trap}}{\bar{M} g} \tag{12}$$

Here, b_{i-j} (mol/m/s) is the binary diffusion coefficient of the i-th species through the j-th species [17, 18]. These are weighted by the thermosphere mixing ratios of each non-condensible constituent (CO, N_2, and atomic O), which are obtained from the upper atmosphere mixing ratios, and from assuming that all H_2O, H_2, O_2, and CO_2 is photodissociated to CO, O and H (N_2 background is fixed); the mixing ratio of atomic hydrogen, f_H, is similarly calculated (see below). The scale height of hydrogen, H_H (m), and of the background gases, H_n (m), depend on upper atmosphere temperature, $T_{cold-trap}$. The diffusion limited escape flux is φ_{diff} (mol H/m^2/s).

To calculate XUV-driven hydrodynamic escape of H, and associated O and CO_2 drag, we follow Odert et al. [19] and Zahnle and Kasting [18]. The total XUV-energy mass loss rate, Φ_{XUV} (kg/m^2/s) is specified by the following equation:

$$\Phi_{XUV} = \frac{\varepsilon(F_{XUV}, X_O, \zeta, \varepsilon_{lowXUV}) F_{XUV} r_p}{4GM_p} \tag{13}$$

Here, F_{XUV} is the XUV flux (W/m^2) received from the host star. The efficiency of hydrodynamic escape, ε, is a function of atmospheric composition and XUV stellar flux [20]. In general, the XUV-driven mass flux will be partitioned between H loss, O drag and, under very high XUV fluxes, CO drag [19, 20]. For example, the oxygen fractionation factor, χ_O, can be obtained:

$$\chi_O = 1 - \frac{g(m_O - m_H) b_{H-O}}{\Phi_H k_B T_{thermo} (1 + X_O)} \tag{14}$$

Here, T_{thermo} is the thermosphere temperature described in the main text, k_B is the Boltzmann constant, m_i (kg) is the mass of the i-th species, X_O is the upper atmosphere mixing ratio of oxygen. The hydrogen escape flux, Φ_H (molecules H/m^2/s), can be obtained by analytically solving equations (4), (5), and (6) in Odert et al. [19].

Note that we do not explicitly track changing relative abundances of atmospheric constituents above the homopause. Instead, molecular mixing ratios above the cold trap are converted to atomic mixing ratios as follows:

$$f_H = \frac{2 f_{H_2O} + 2 f_{H_2} + 4 f_{CH_4}}{3 f_{H_2O} + 2 f_{H_2} + 4 f_{CH_4} + 2 f_{O_2} + 2 f_{CO_2} + f_{CO} + f_{N_2}} \tag{15}$$

$$f_O = \frac{f_{H_2O} + 2f_{O_2} + f_{CO_2}}{3f_{H_2O} + 2f_{H_2} + 4f_{CH_4} + 2f_{O_2} + 2f_{CO_2} + f_{CO} + f_{N_2}} \quad (16)$$

We assume that CO_2 is dissociated to CO and O, CO is robust against photodissociation, and that CH_4 can be treated as equivalent to CO for the purposes of estimating hydrodynamic loss of C. This means that the upper atmosphere CO abundance is given by:

$$f_{CO} = \frac{f_{CO} + f_{CO_2} + f_{CH_4}}{3f_{H_2O} + 2f_{H_2} + 4f_{CH_4} + 2f_{O_2} + 2f_{CO_2} + f_{CO} + f_{N_2}} \quad (17)$$

This approach is implicitly pessimistic about secondary atmosphere retention since we are effectively assuming well-mixed constituents all the way up to the exobase; this maximizes the abundance of high molecular weight constituents vulnerable to XUV-driven hydrodynamic drag. Accounting for the sedimentation of heavier species would only lower the amount of C and O that could be dragged to space.

The combination of diffusion-limited and XUV-limited thermal escape parameterizations ensures diffusion-limited H escape for low stratospheric abundances, and a smoothly transition to XUV-driven escape as the upper atmosphere becomes steam or H_2 dominated [20]. The precise transition abundance is unknown and will, in general, depend on conductive and radiative cooling of the upper atmosphere as well as downward diffusive transport. Here, it is represented by the free parameter, λ_{tra}, which ranges from 10^{-6} to 10^{1} and is sampled uniformly in log space (corresponding to transition H mixing ratios from ~0.006 to 0.6). The efficiency of hydrodynamic escape is parameterized by loosely following the approach of Wordsworth et al. [21]. If the XUV-stellar flux is insufficient to drag oxygen, then the efficiency is equal to a constant, ε_{lowXUV}, which is randomly sampled from 1% to 30%. Alternatively, if the XUV-stellar flux exceeds what is required to drag O, then some portion of the excess energy, ζ_{high}, goes into driving further escape, whereas the rest, $1-\zeta_{high}$, is assumed to be efficiently radiated away. The efficiency factor, ζ_{high}, is randomly sampled from 0-100% for complete generality. See Krissansen-Totton et al. [20] for analytic expressions. We focus on XUV-driven mass loss rather than core-powered mass loss since this is expected to be the dominant mechanism for habitable zone, Earth-sized planets [22].

Fig. S5 illustrates example Monte Carlo stellar evolution and escape calculations. The top row of Fig S5 shows the input TRAPPIST-1 bolometric luminosity evolution and XUV luminosity evolution [23], respectively. The middle row shows show escape rates of H and O, respectively, for TRAPPIST-1b where an H-rich upper atmosphere is assumed (mixing ratio of atomic species, XH = 0.6, XO = 0.2, and XCO = 0.2). The bottom row of Fig S5 shows escape rates of H and O, respectively, for TRAPPIST-1b where an H-poor upper atmosphere is assumed (mixing ratio of atomic species, XH = 0.001, XO = 0.2, and XCO = 0.799). The individual lines represent luminosities and escape fluxes sampled from the full Monte Carlo ranges described in Table 1.

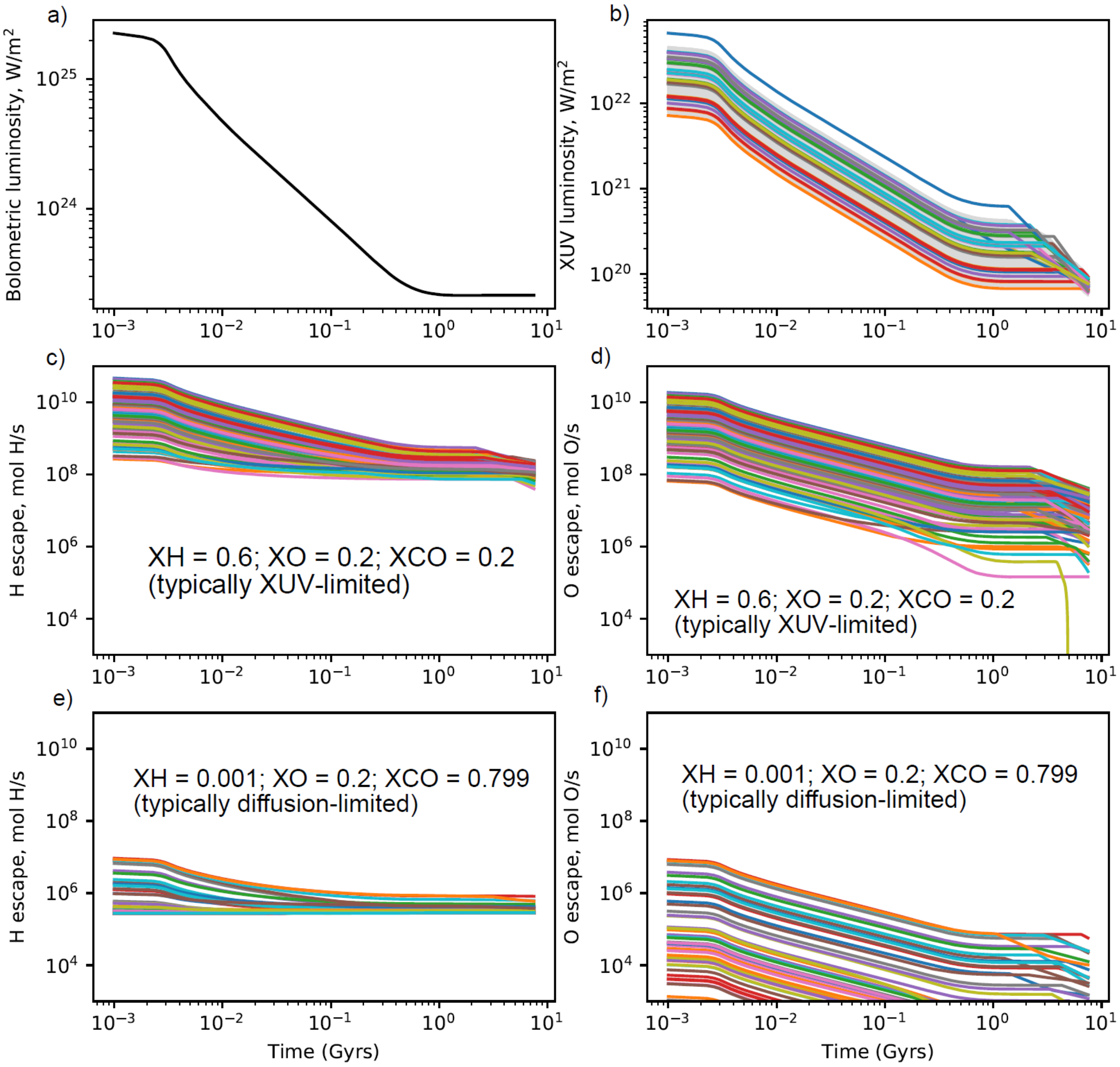

Fig. S5: Illustrative stellar evolution and escape calculations showing Monte Carlo spread. Subplot (a) shows input bolometric luminosity evolution of TRAPPIST-1, and (b) shows sampled input XUV luminosity evolution. Subplot (c) and (d) show H and O escape fluxes through time, respectively, for a H-rich atmosphere (XUV-limited regime) whereas (e) and (f) show H and O escape fluxes, respectively, for a H-poor atmosphere (diffusion-limited regime). For these illustrative calculations atmospheric composition is held constant to show the spread in fluxes as stellar luminosity evolves in time.

Sensitivity Tests and Additional Results

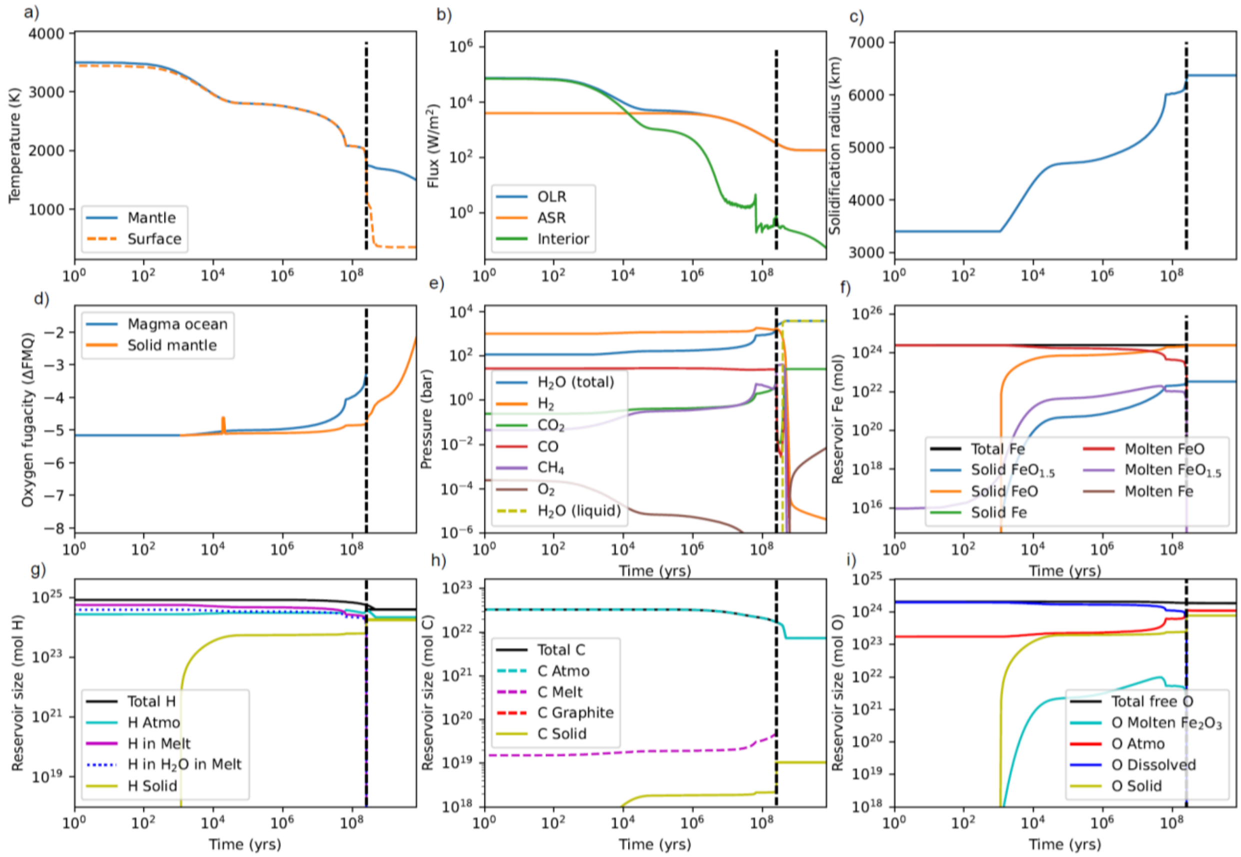

Fig. S6: Identical to Fig. 3 in the main text showing TRAPPIST-1e with a large initial H endowment, but in this case we make the alternative endmember assumption whereby all metallic iron produced by hydrogen reducing FeO (H_2+FeO -> Fe + H_2O) is instantly sequestered in the metallic core. Subplot (a) denotes the time-evolution of surface (orange) and mantle potential temperature (blue), (b) denotes the evolution of outgoing longwave radiation (OLR, blue), absorbed shortwave radiation (ASR, orange), and interior heatflow (green), and subplot (c) shows the evolution of the magma ocean solidification front from the core-mantle boundary to the surface. Subplot (d) shows solid mantle (orange) and magma ocean redox (blue) relative to the Fayalite-Quartz-Magnetite (FMQ) buffer, (e) shows the evolution of atmospheric composition including H_2O, H_2, CO_2, CO, CH_4, and O_2. Subplot (f) denotes iron speciation in both the magma ocean and the solid silicate mantle. Subplot (g) shows both solid and fluid reservoirs of H including total dissolved hydrogen (purple) and hydrogen dissolved as H_2O (blue-dotted). Subplot (h) denotes solid and fluid reservoirs of C and subplot (i) denotes solid and fluid reservoirs of free oxygen, including oxygen bound to ferric iron, atmospheric species, and O in volatiles dissolved in the melt (H_2O, CO_2) reservoirs respectively. Vertical dashed black lines show the termination of the magma ocean.

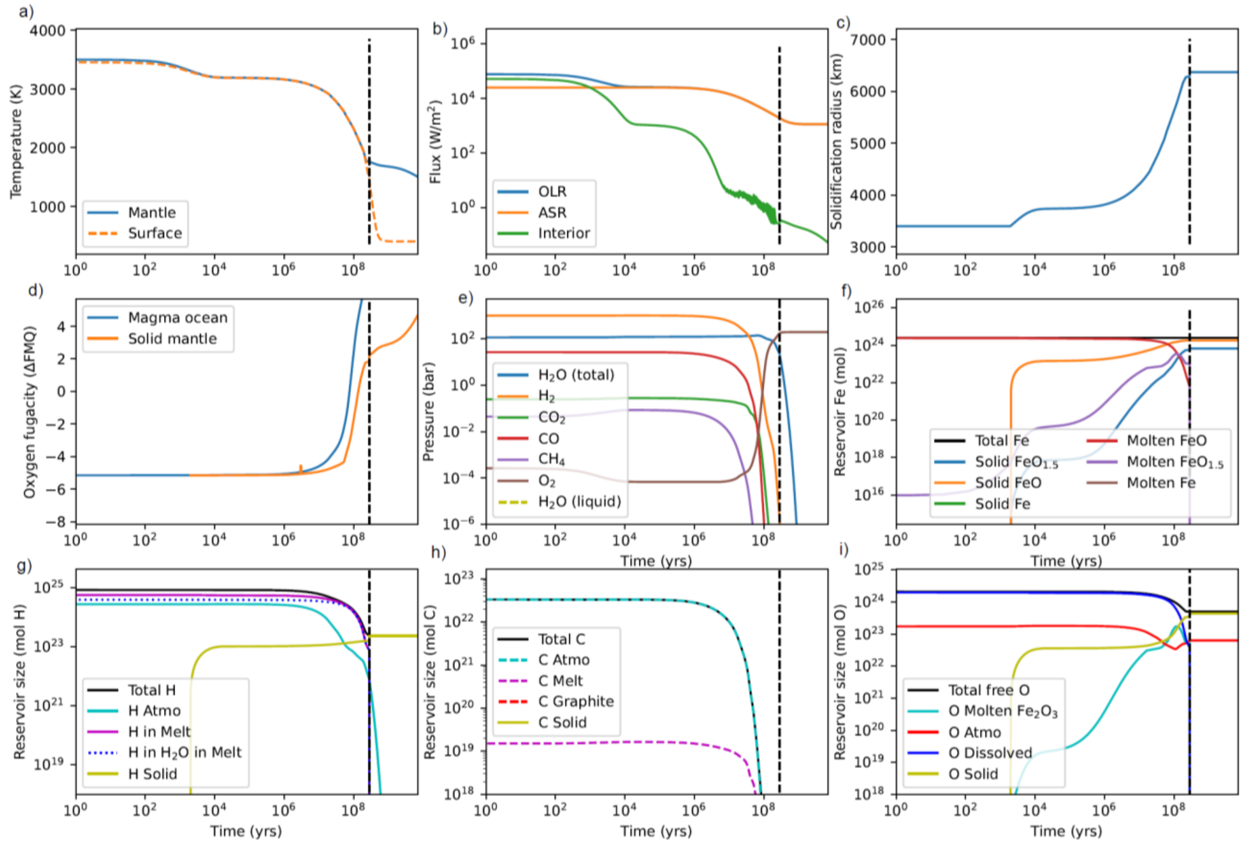

Fig. S7: Identical to Fig. 4 in the main text showing TRAPPIST-1b with a large initial H endowment, but in this case, we make the alternative endmember assumption whereby all metallic iron produced by hydrogen reducing FeO (H_2+FeO -> Fe + H_2O) is instantly sequestered in the metallic core. Subplot (a) denotes the time-evolution of surface (orange) and mantle potential temperature (blue), (b) denotes the evolution of outgoing longwave radiation (OLR, blue), absorbed shortwave radiation (ASR, orange), and interior heatflow (green), and subplot (c) shows the evolution of the magma ocean solidification front from the core-mantle boundary to the surface. Subplot (d) shows solid mantle (orange) and magma ocean redox (blue) relative to the Fayalite-Quartz-Magnetite (FMQ) buffer—the final surface is expected to be highly oxidized—(e) shows the evolution of atmospheric composition including H_2O, H_2, CO_2, CO, CH_4, and O_2. Subplot (f) denotes iron speciation in both the magma ocean and the solid silicate mantle. Subplot (g) shows both solid and fluid reservoirs of H including total dissolved hydrogen (purple) and hydrogen dissolved as H_2O (blue-dotted). Subplot (h) denotes solid and fluid reservoirs of C and subplot (i) denotes solid and fluid reservoirs of free oxygen, including oxygen bound to ferric iron, atmospheric species, and O in volatiles dissolved in the melt (H_2O, CO_2) reservoirs respectively. Vertical dashed black lines show the termination of the magma ocean.

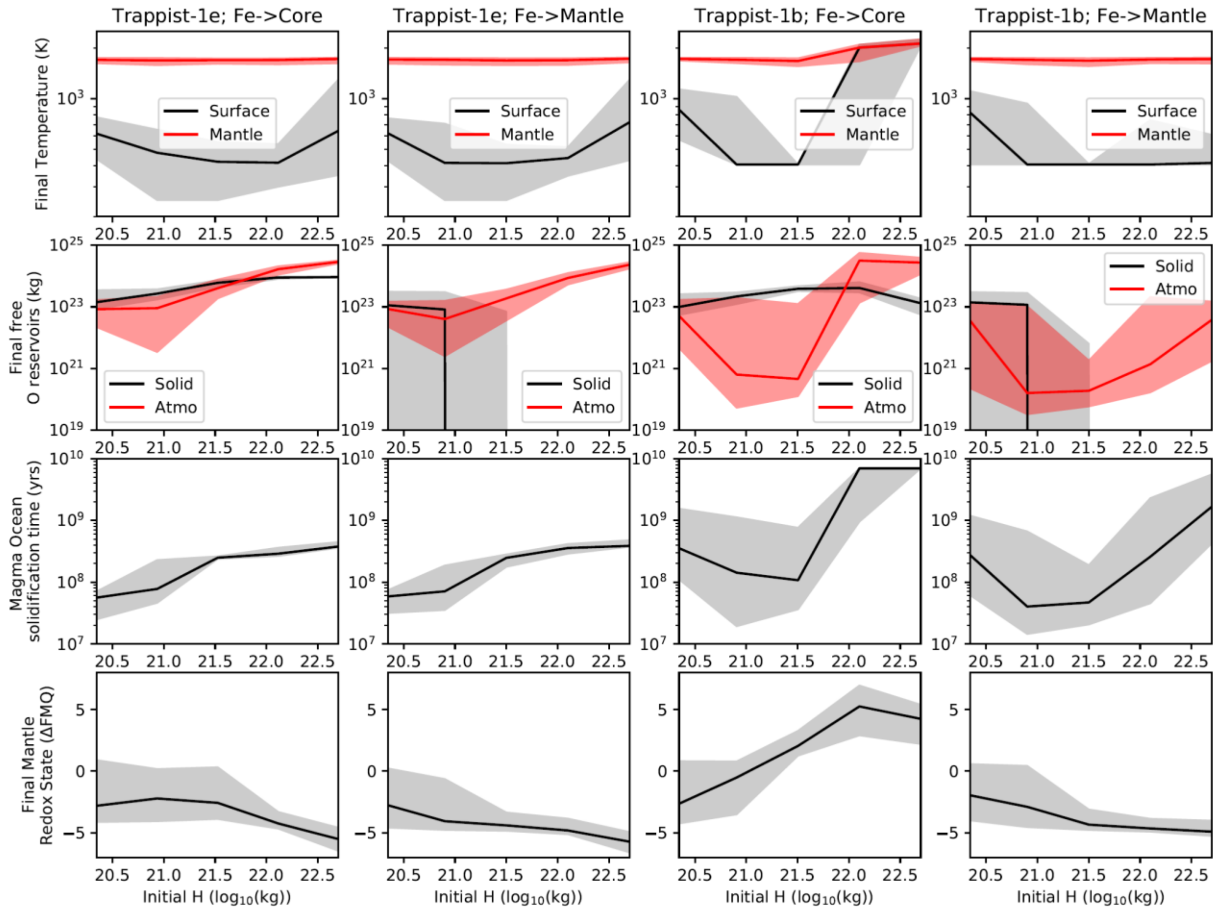

Fig. S8: Additional outputs from Monte Carlo calculations shown in Fig. 5 comparing outcomes for TRAPPIST 1e (left two columns) and TRAPPIST-1b (right two columns) after 8 Gyr of coupled atmosphere-interior-redox evolution, as a function of initial H endowment. Rows denote final mantle and surface temperatures (K), final atmosphere and interior free oxygen inventories (kg), the time taken for magma ocean solidification (years), and final mantle redox state relative to FMQ, respectively. The two columns for each planet denote endmember cases whereby all metallic iron is sequestered in the core (left), and all metallic iron remains in the silicate mantle (right). Solid lines and shaded regions denote median model outputs and 1-sigma confidence intervals, respectively. The negative free oxygen abundances indicate metallic iron exceeds ferric iron in the mantle (ferrous iron has zero free oxygen, metallic iron is effectively negative free oxygen – see methods). The magma ocean never solidifies in some cases for TRAPPIST-1b i.e. the solidification time equals the 8 Gyr age of the system. Finally, the final mantle redox state of TRAPPIST-1b may reflect whether metallic iron was sequestered in the core or remains in the mantle.

Sensitivity to Planet Mass

In the nominal calculations, the mass and radii of TRAPPIST-1b and e were assumed to equal one Earth mass (and one Earth radii) respectively, to isolate the effects of differing instellation histories. Here, we perform a sensitivity test with the true mass and radii of each planet [24]. Results are shown in Fig S9 and are very similar to nominal calculations.

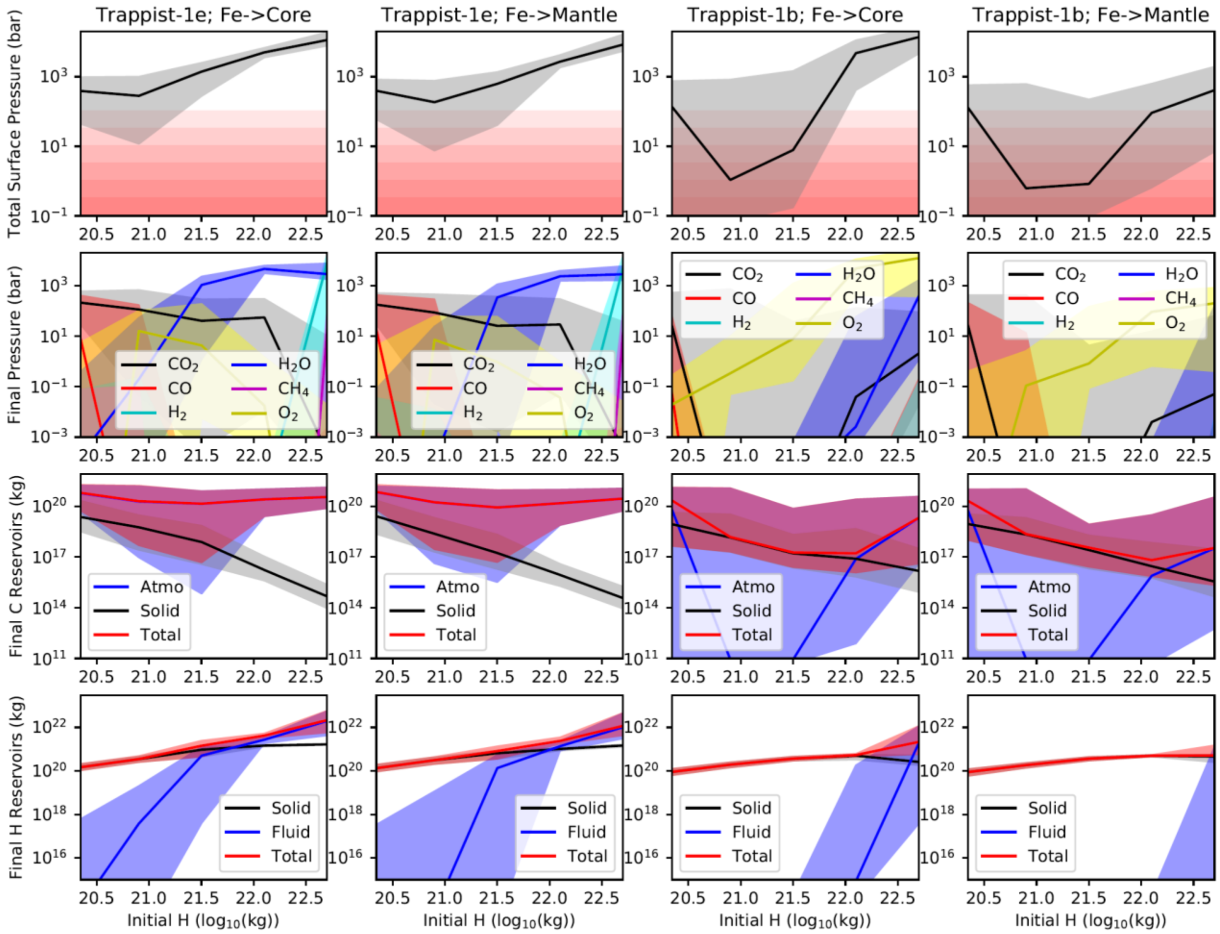

Fig. S9: Identical to Fig. 5 in the main text except that the true mass and radii of TRAPPIST-1e and b are assumed [24].

Sensitivity to initial mantle FeO

In all nominal calculations in the main text, an Earth-like mantle FeO fraction was assumed (mole fraction 0.06). We repeated all nominal calculations with both smaller (0.02) and larger (0.2) initial FeO mole fractions to test sensitivity to this assumption. This range encompasses the range of silicate mantle iron content for solar system bodies, and differentiated exoplanets are likely to inherit similar compositions given expect silicate-metal elemental partitioning [25] and the muted variability in stellar rock-forming element abundances [26]. Fig. S10 shows the low initial iron case. Broadly speaking, results are similar to nominal calculations because there is still plenty of FeO available to be reduced. Fig. S11 shows the high initial FeO case, which results in somewhat higher final surface water inventories for TRAPPIST-1e since more iron oxide is available to be reduced. For TRAPPIST-1b, results are qualitatively similar to nominal calculations.

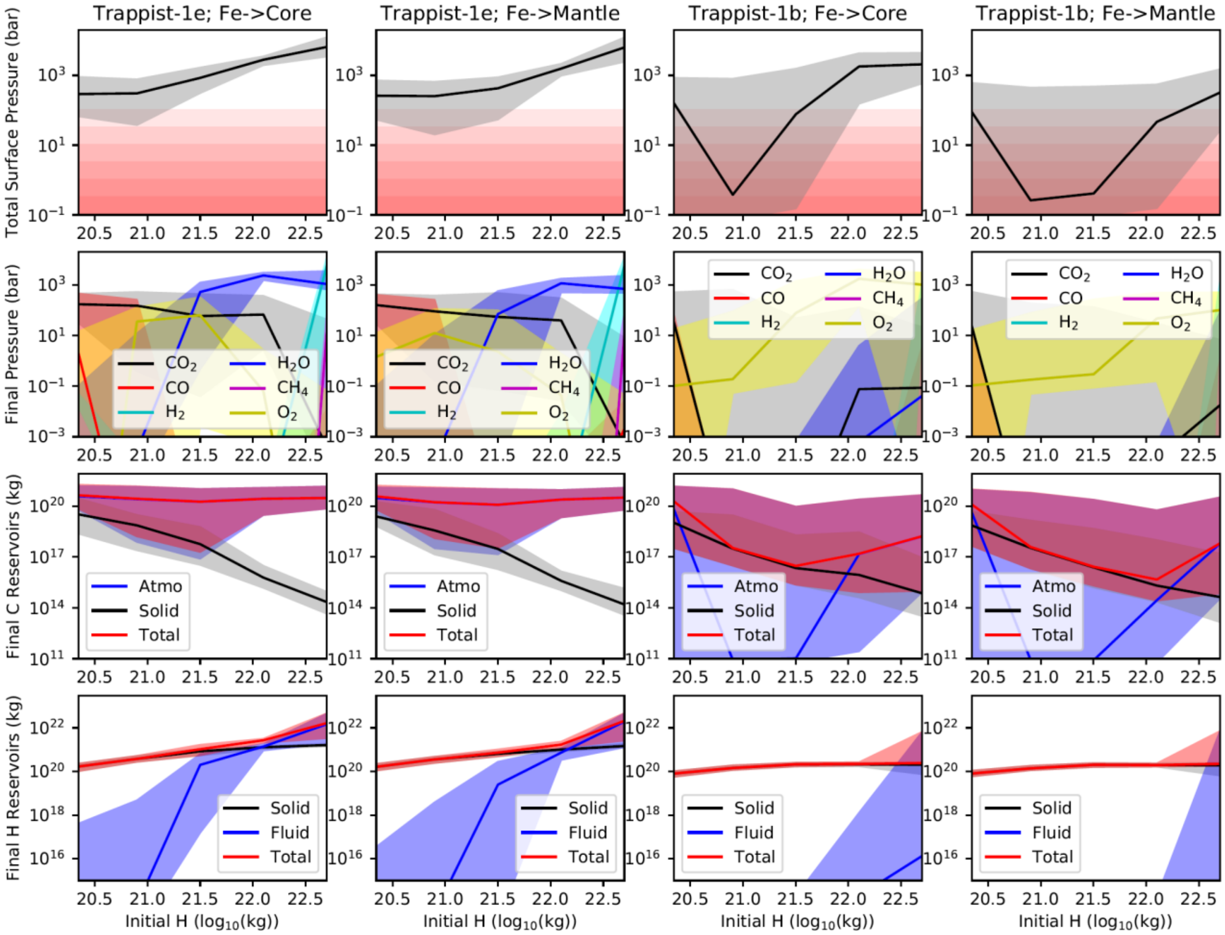

Fig. S10: Identical to Fig. 5 in the main text except that the initial mantle iron oxide content is three times smaller than that of the Earth.

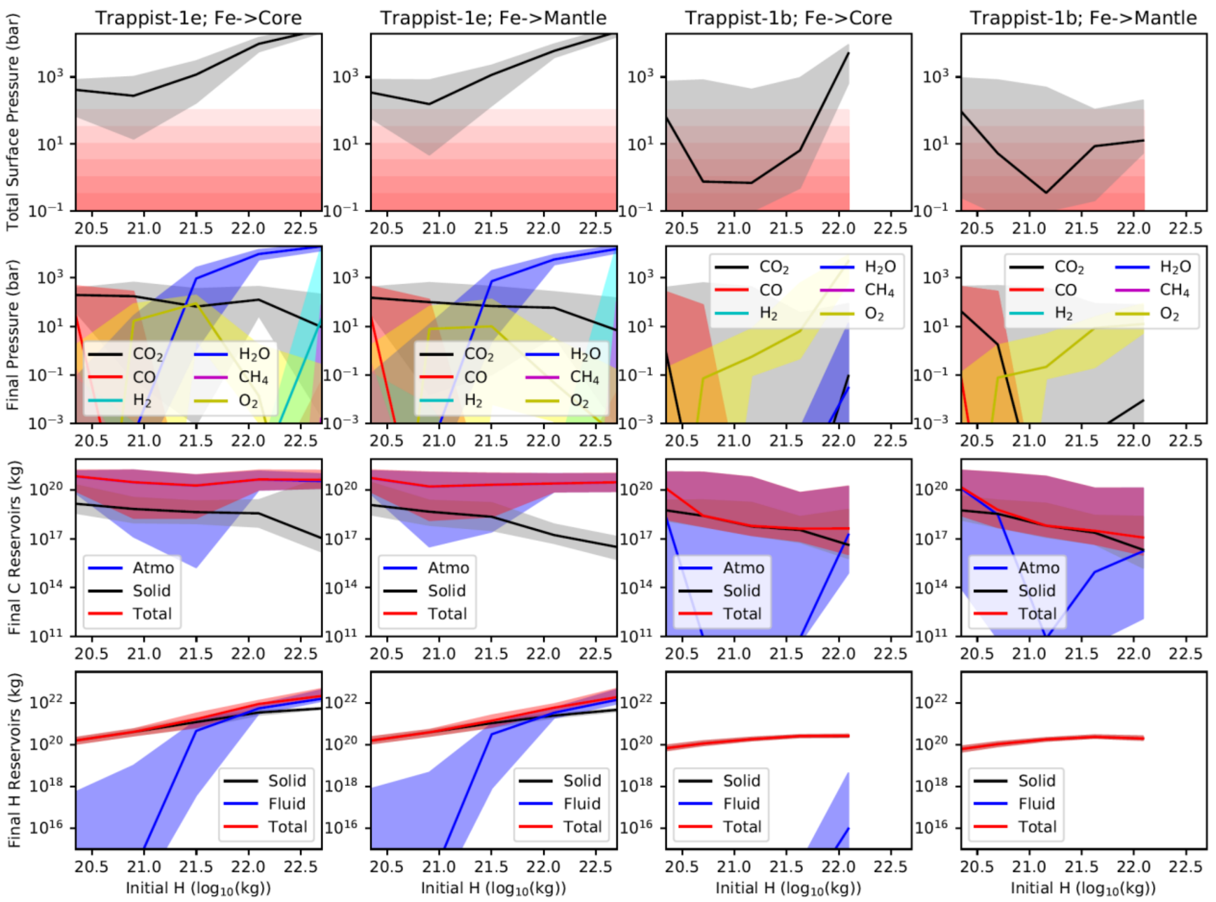

Fig. S11: Identical to Fig. 5 in the main text except that the initial mantle iron oxide content is three times greater than that of the Earth. Note that model failure rates are higher for high initial H due to the very large surface volatile inventories that are generated.

Sensitivity to atmospheric structure

In all nominal calculations in the main text, an adiabatic deep atmosphere is assumed, which neglects the possibility of a transition to a deep radiative zone [27]. We thus recomputed our climate grid assuming an isothermal atmospheric structure below 100 bar to bookend the two plausible endmember cases for the deep atmosphere (adiabat vs. isotherm). Fig. S12 and S13 show the results of these calculations for TRAPPIST-1e. Unsurprisingly, an isothermal deep atmosphere leads to cooler surface temperatures and shorter duration magma oceans (Fig S13). However, overall qualitative outcomes for TRAPPIST-1e are similar to the nominal model because the escape fluxes are most strongly influenced by luminosity evolution and the runaway greenhouse duration, neither of which are changed by assuming a deep isothermal atmosphere. Hydrogen-rich atmospheres are slightly more likely to be retained for the cooler (deep isotherm) cases.

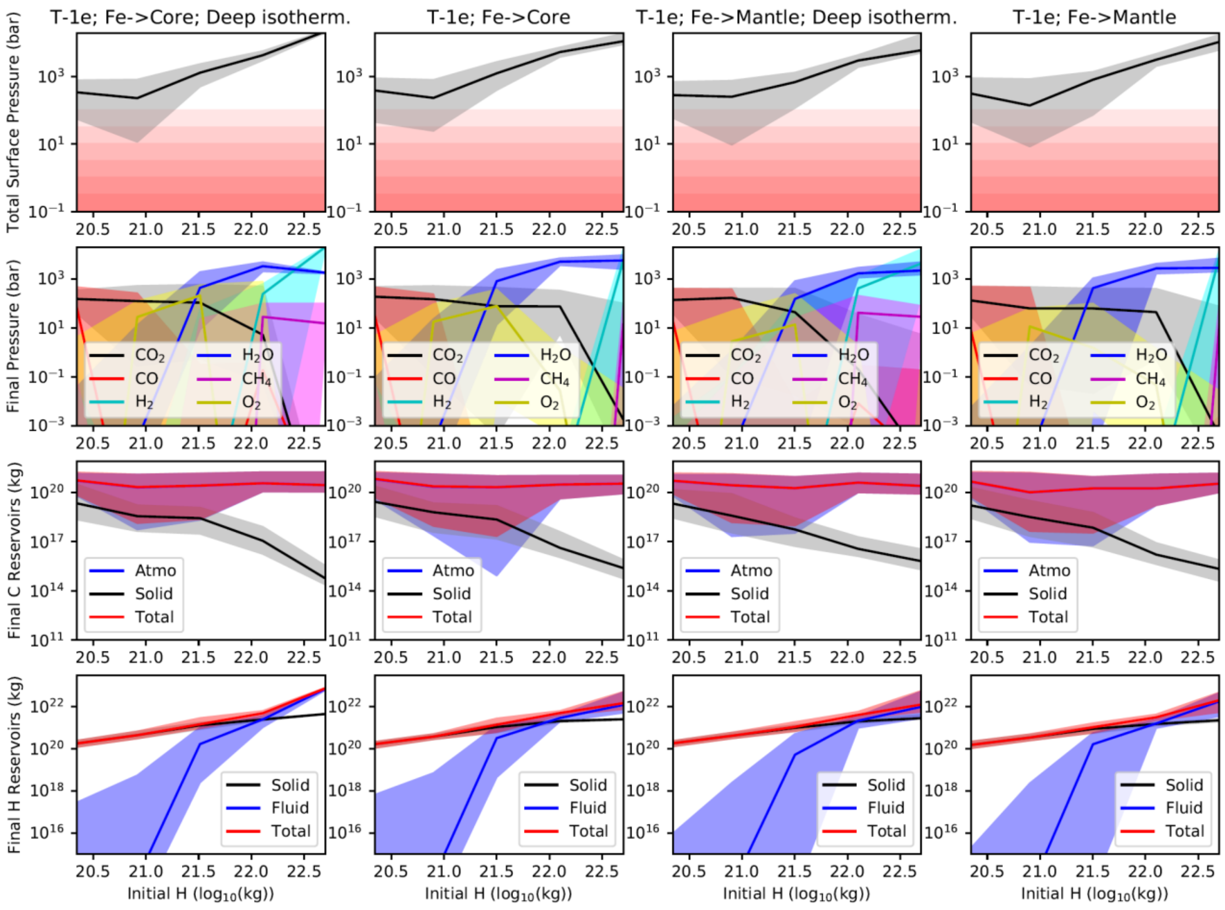

Fig. S12: Calculations repeated for TRAPPIST-1e using a climate model with a deep isotherm (below 100 bar) to simulate a radiative deep atmosphere. Results are qualitatively similar to nominal calculations because escape is primarily governed by stellar bolometric and XUV evolution, and not by the temperature evolution of the atmosphere-interior interface.

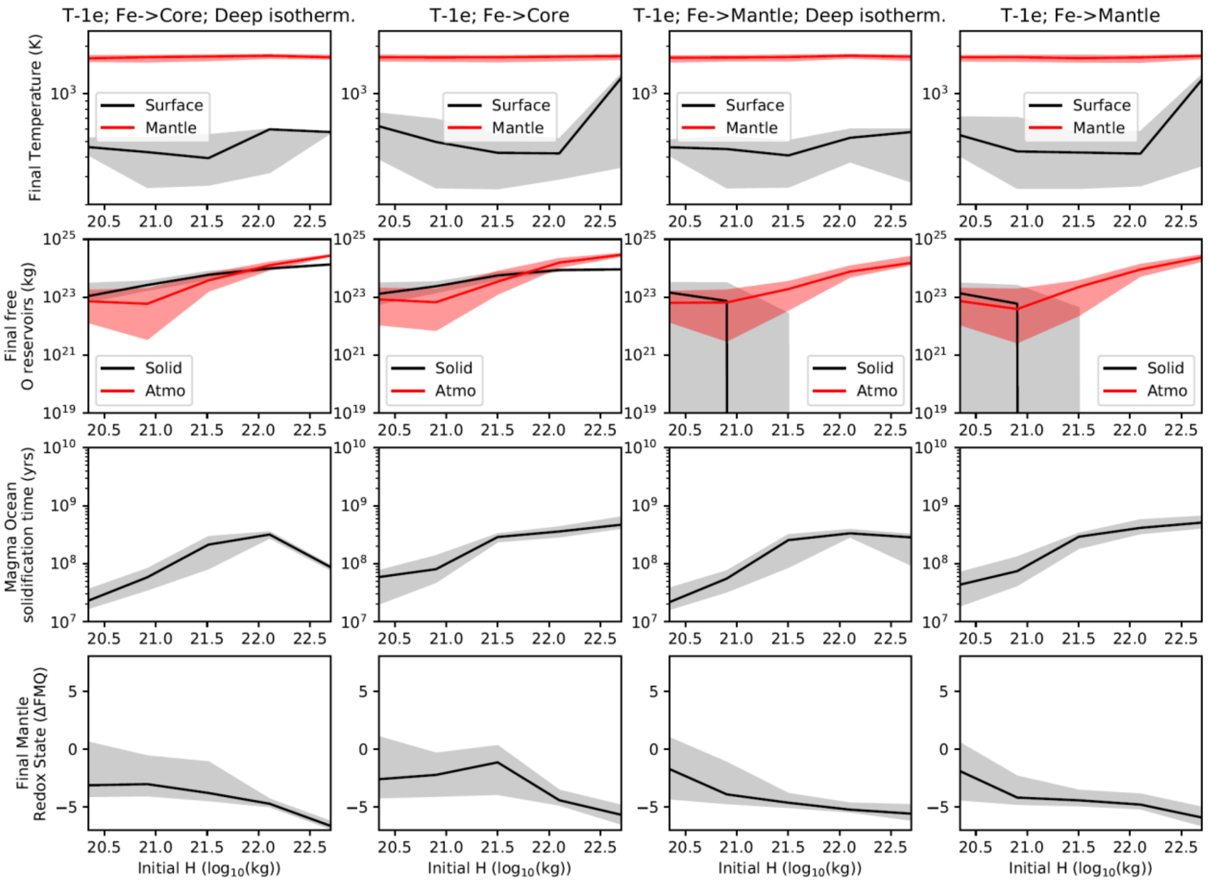

Fig. S13: Additional outputs from deep isotherm sensitivity test shown in Fig. S12 comparing outcomes for TRAPPIST 1e. A radiative deep atmosphere results in cooler surface temperatures and shorter duration magma oceans compared to nominal calculations.

Sensitivity to clouds

In all nominal calculations in the main text a low albedo is assumed to represent the cloud-free magma ocean state (values between 0 and 0.2 are sampled in Monte Carlo calculations). This maximizes the duration of the runaway greenhouse compared to cloudy scenarios, and by extension the longevity of hydrodynamic escape. However, to test the effect of albedo on our results we repeated calculations using a higher albedo range (0.5-0.7) to represent continuously cloudy or hazy atmospheres, as shown in Fig. S14 and S15. While surface temperatures are slightly cooler and the typical duration of the magma ocean is a factor of a few shorter for the high albedo cases, atmospheric composition outcomes are largely unchanged.

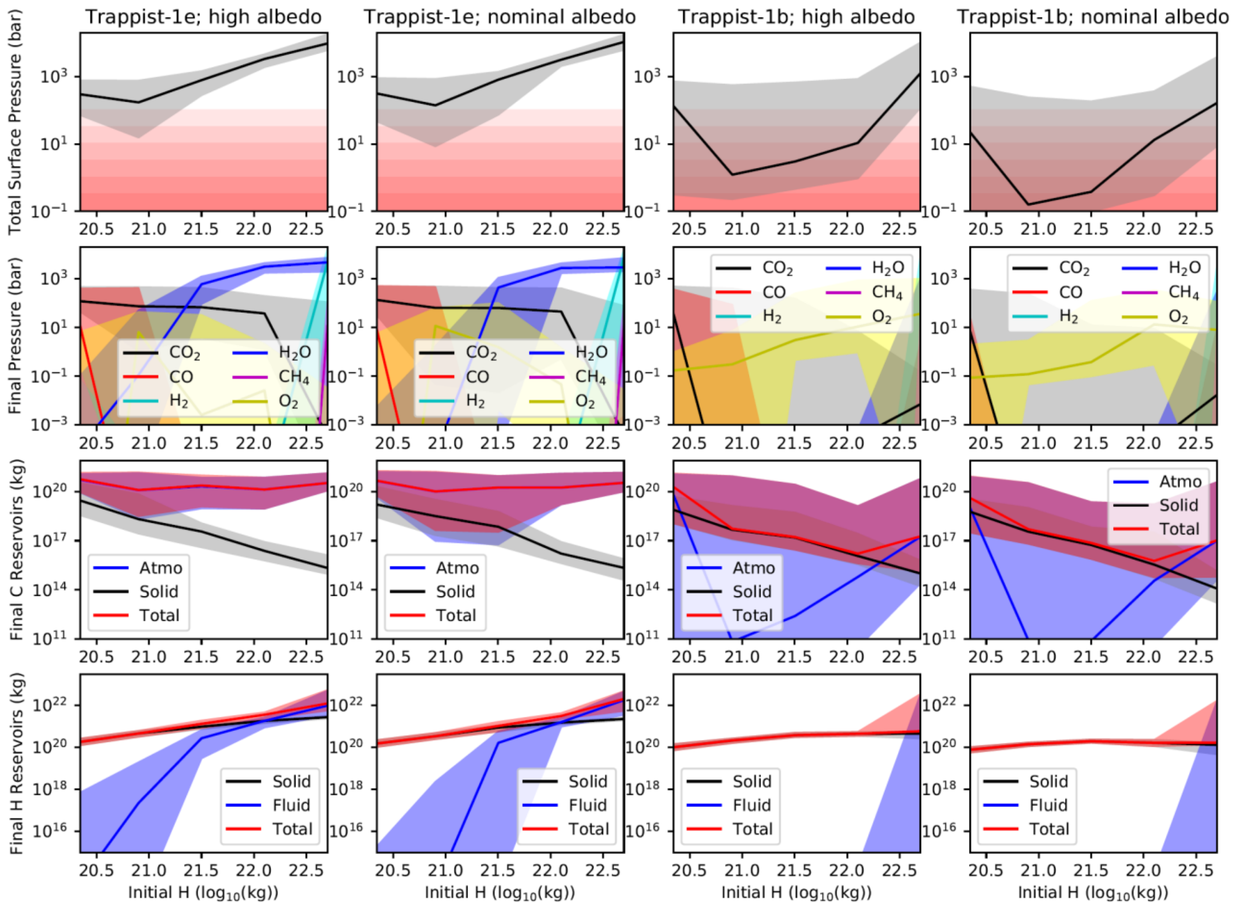

Fig. S14: Calculations repeated for TRAPPIST-1e and b but with a higher albedo range (0.5-0.7) compared to low albedos sampled in the nominal calculations (0-0.2). Slightly less C is lost in the cooler, high albedo cases, but results are qualitatively very similar to nominal calculations.

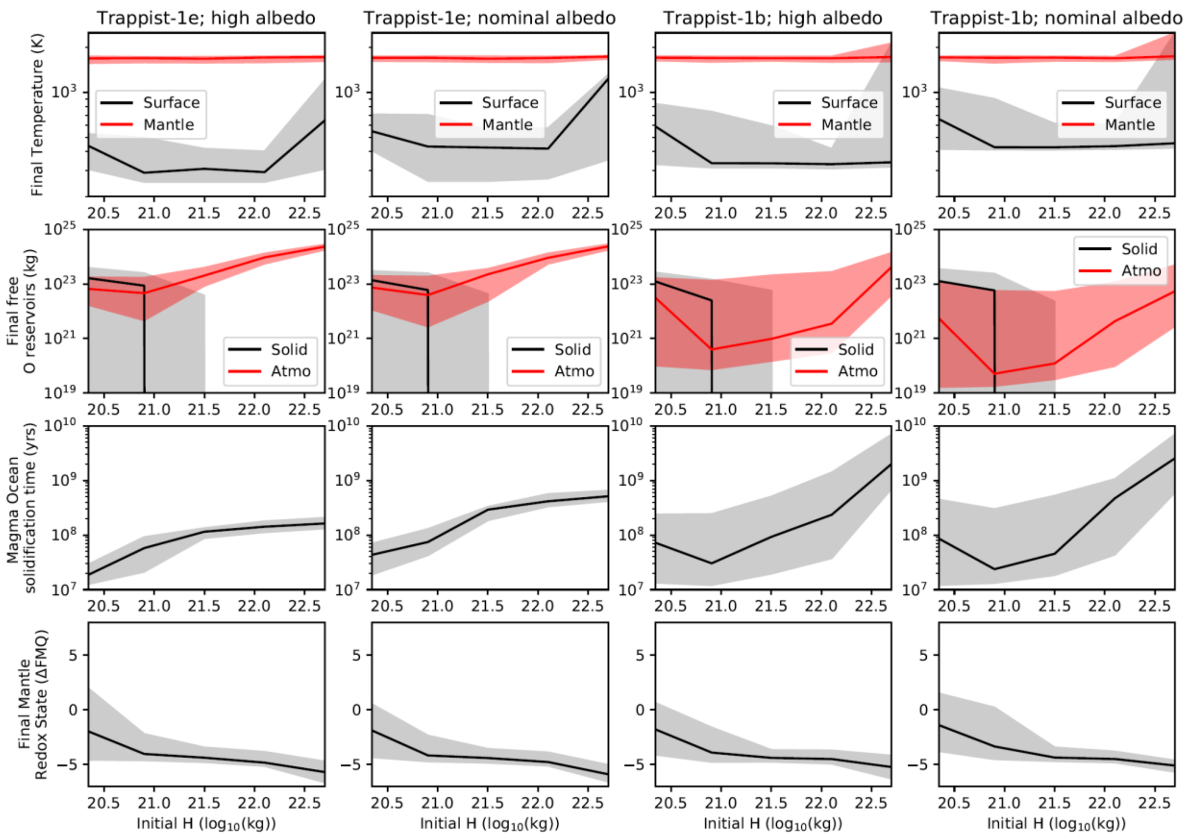

Fig. S15: Additional outputs from high albedo sensitivity test shown in Fig. S14 comparing outcomes for TRAPPIST 1e and b. Surface temperatures are cooler, and the typical duration of the magma ocean is a factor of a few smaller for the high albedo cases, but this is not sufficient to dramatically change atmosphere-interior evolution.

Sensitivity to melt trapping

In all nominal calculations in the main text, melt is trapped in the solidifying mantle using the parameterization described in Krissansen-Totton and Fortney [28] and Hier-Majumder and Hirschmann [2], which does not permit trapped melt fractions above 0.3. This may potentially underestimate the amount of volatiles trapped inside the mantle if the rheological transition occurs at higher melt fractions [29], or if melt concentrations vary as a function of melt fraction [30]. To test the influence of greater melt trapping at higher solidification rates, we increased the maximum trapped melt fraction to 0.8 and repeated calculations for TRAPPIST-1b and e (Fig. S16). This does not substantially change atmospheric outcomes since the pace of magma ocean solidification is determined by stellar bolometric luminosity evolution and is typically slow. Because compaction is rapid compared to solidification timescales, equilibration of volatiles between melt phases and the atmosphere will occur.

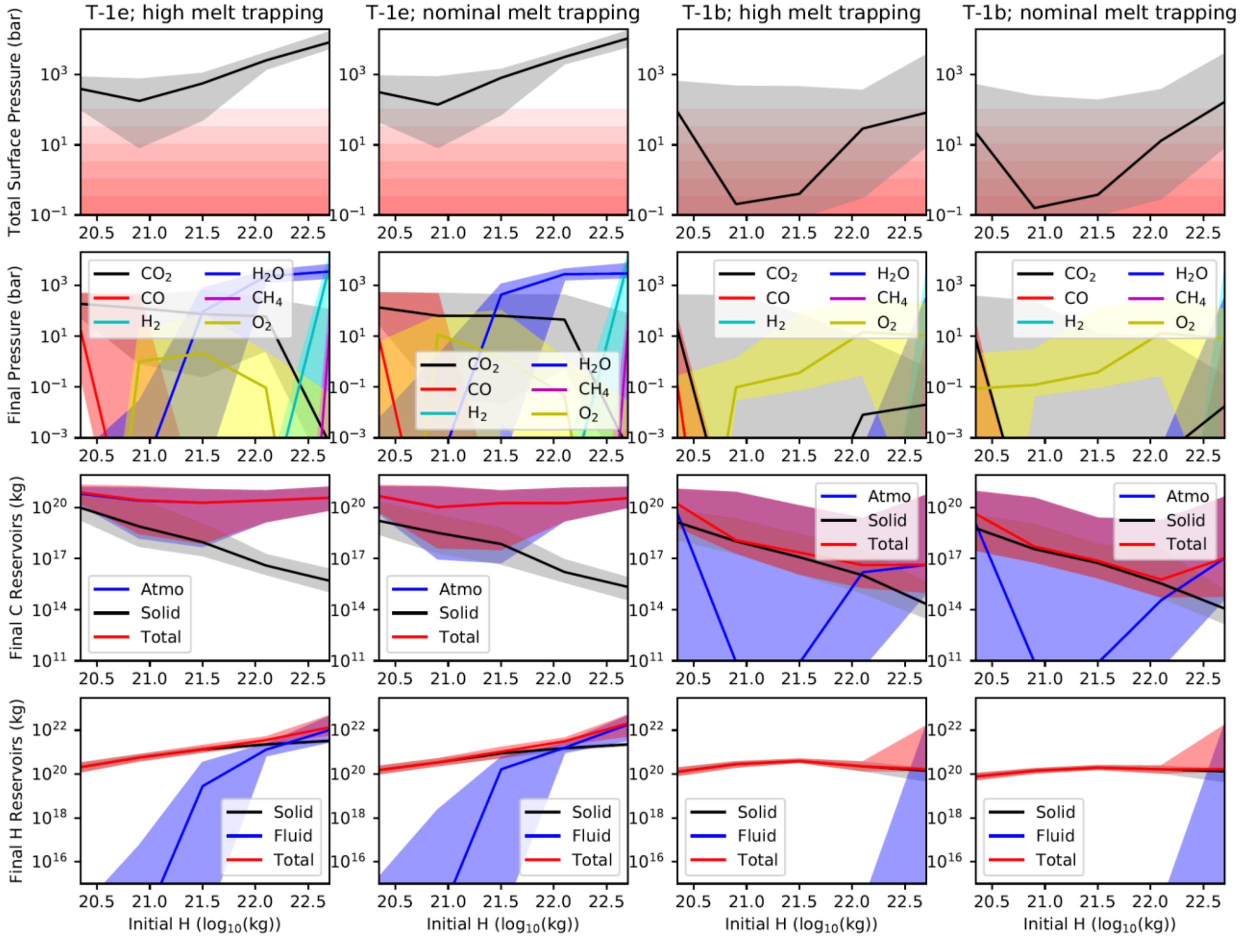

Fig. S16: Calculations repeated for TRAPPIST-1e and b but with a higher maximum trapped melt fraction (0.8) compared to nominal calculations (0.3). Results are largely insensitive to melt trapping assumptions, except that slightly more C is retained in the mantle for fast solidification (low initial H) model runs.

Application to other planetary systems

To establish our results are not specific to the TRAPPIST-1 system, we repeated calculations for LP 890-9 (Fig. S17) and Proxima Centauri (Fig. S18). The LP 890-9 system [31] contains a highly irradiated super Earth, b (1.32R$_\oplus$, assumed mass 2.3 M$_\oplus$), and a potentially habitable super Earth, c (1.37 R$_\oplus$, assumed mass 2.5 M$_\oplus$) orbiting an M6 star. Proxima Centauri b is an Earth-sized (1.07 M$_\oplus$, assumed radius 1.2 R$_\oplus$) orbiting within the habitable zone (0.65 S$_\oplus$) of its M5 host [32]. Calculations for TRAPPIST-1f are also included to show the potential impact of lowering equilibrium temperature. For all planets, bolometric luminosity evolution is interpolated from Baraffe et al. [33] and TRAPPIST-1 XUV evolution [23] is assumed to maximize XUV-driven escape potential. Despite this pessimistic assumption, we find all habitable zone planets are likely to retain substantial inventories of surface water for initial hydrogen endowments greater than BSE (analogous to TRAPPIST-1e in the main text). Similarly, the Venus analog LP 890-9 b is far more likely to have stripped of all surface volatiles, although note that this is slightly less likely than for TRAPPIST-1b owing to LP 890-9 b's higher gravity.

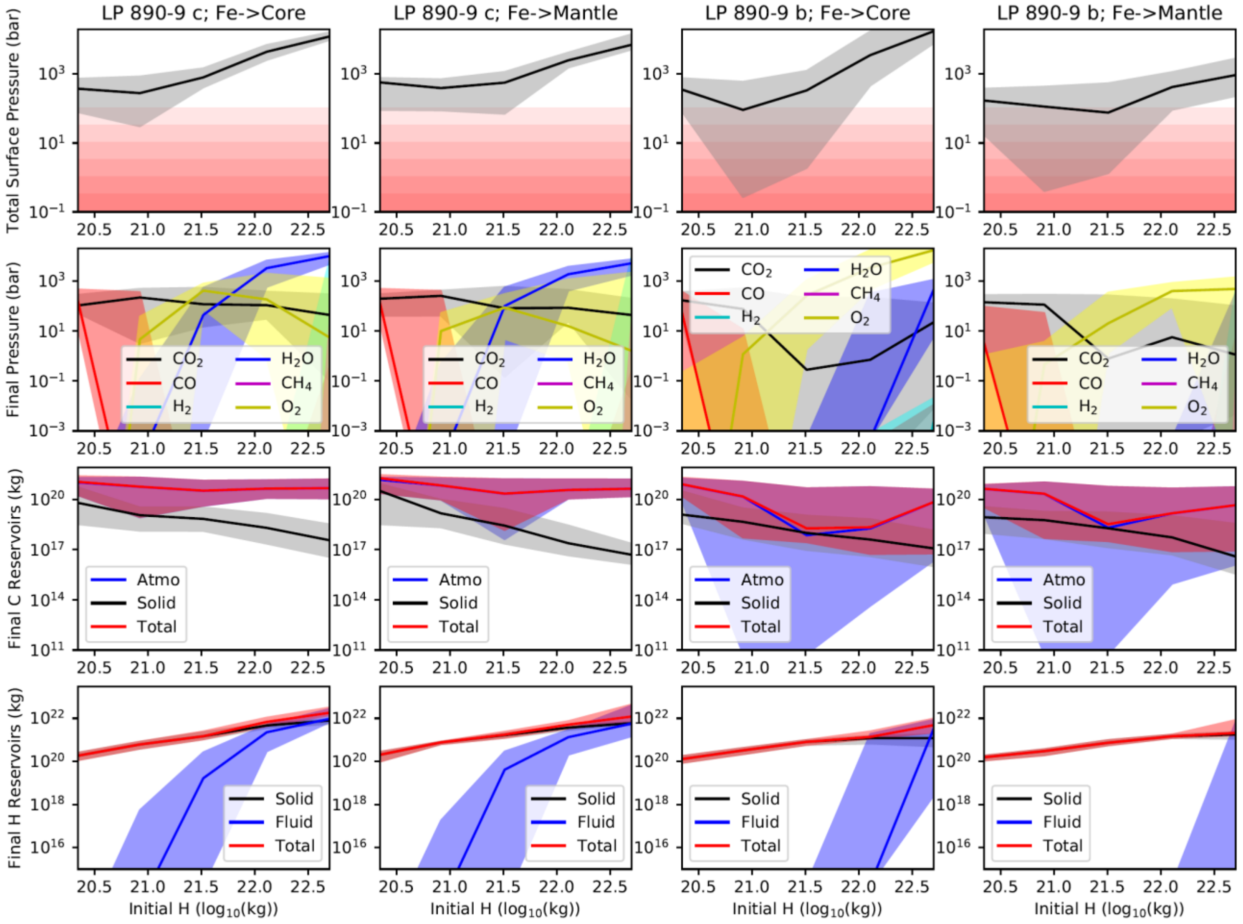

Fig. S17: Identical to Fig. 5 in the main text except calculations are applied to the potentially habitable 0.906 ± 0.026 S$_\oplus$) LP 890-9 c (left) and the highly irradiated (4.09±0.12 S$_\oplus$) LP 890-9 b (right).

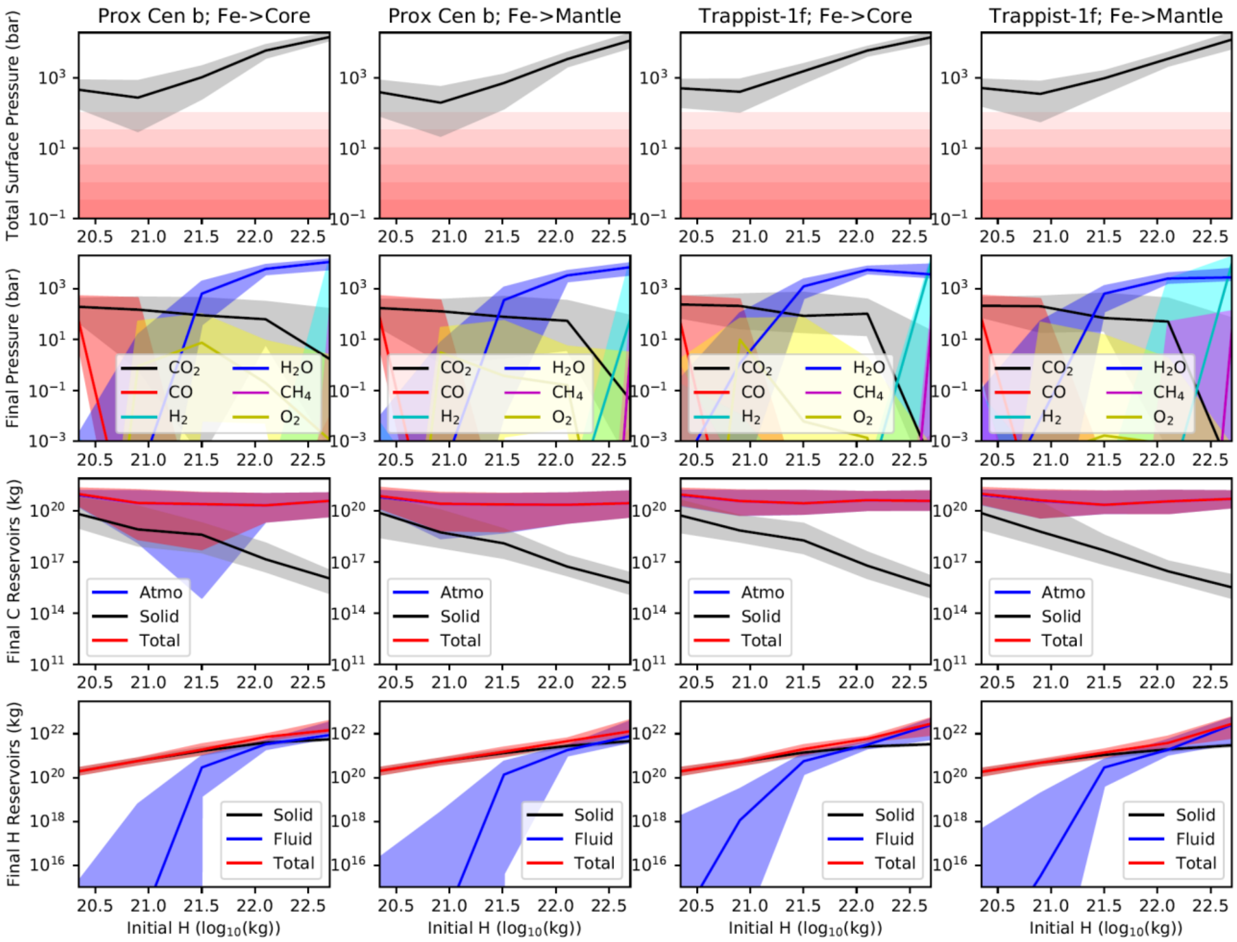

Fig. S18: Identical to Fig. 5 in the main text except calculations are applied to the habitable zone terrestrial planets Proxima Centauri b (left) and TRAPPIST-1f (right).

Supplementary References

1. Miyazaki, Y. and J. Korenaga, *Inefficient water degassing inhibits ocean formation on rocky planets: An insight from self-consistent mantle degassing models.* Astrobiology, 2022. **22**(6): p. 713-734.
2. Hier-Majumder, S. and M.M. Hirschmann, *The origin of volatiles in the E arth's mantle.* Geochemistry, Geophysics, Geosystems, 2017. **18**(8): p. 3078-3092.
3. Bower, D.J., et al., *Retention of water in terrestrial magma oceans and carbon-rich early atmospheres.* The planetary science journal, 2022. **3**(4): p. 93.
4. Sossi, P.A., et al., *Solubility of water in peridotite liquids and the prevalence of steam atmospheres on rocky planets.* Earth and Planetary Science Letters, 2023. **601**: p. 117894.
5. Kadoya, S., J. Krissansen-Totton, and D.C. Catling, *Probable cold and alkaline surface environment of the Hadean Earth caused by impact ejecta weathering.* Geochemistry, Geophysics, Geosystems, 2020. **21**(1): p. e2019GC008734.

6. Sleep, N.H. and K. Zahnle, *Carbon dioxide cycling and implications for climate on ancient Earth.* Journal of Geophysical Research: Planets, 2001. **106**(E1): p. 1373-1399.
7. Hamano, K., Y. Abe, and H. Genda, *Emergence of two types of terrestrial planet on solidification of magma ocean.* Nature, 2013. **497**(7451): p. 607-610.
8. Lebrun, T., et al., *Thermal evolution of an early magma ocean in interaction with the atmosphere.* Journal of Geophysical Research: Planets, 2013. **118**(6): p. 1155-1176.
9. Salvador, A., et al., *The relative influence of H2O and CO2 on the primitive surface conditions and evolution of rocky planets.* Journal of Geophysical Research: Planets, 2017. **122**(7): p. 1458-1486.
10. Ortenzi, G., et al., *Mantle redox state drives outgassing chemistry and atmospheric composition of rocky planets.* Scientific Reports, 2020. **10**(1): p. 1-14.
11. Kress, V.C. and I.S. Carmichael, *The compressibility of silicate liquids containing Fe 2 O 3 and the effect of composition, temperature, oxygen fugacity and pressure on their redox states.* Contributions to Mineralogy and Petrology, 1991. **108**: p. 82-92.
12. O'Neill, H., *Cosmochemical estimates of mantle composition*, in *Treatise on Geochemistry: Oxford*. 2014, Elsevier. p. 1-39.
13. Hirschmann, M., *Magma oceans, iron and chromium redox, and the origin of comparatively oxidized planetary mantles.* Geochimica et Cosmochimica Acta, 2022. **328**: p. 221-241.
14. Ma, Z., *Thermodynamic description for concentrated metallic solutions using interaction parameters.* Metallurgical and Materials Transactions B, 2001. **32**: p. 87-103.
15. Frost, B.R., *Introduction to oxygen fugacity and its petrologic importance*, in *Oxide minerals*. 2018, De Gruyter. p. 1-10.
16. Catling, D.C. and J.F. Kasting, *Atmospheric Evolution on Inhabited and Lifeless Worlds*. 2017: Cambridge University Press.
17. Marrero, T.R. and E.A. Mason, *Gaseous diffusion coefficients.* Journal of Physical and Chemical Reference Data, 1972. **1**(1): p. 3-118.
18. Zahnle, K.J. and J.F. Kasting, *Mass fractionation during transonic escape and implications for loss of water from Mars and Venus.* Icarus, 1986. **68**(3): p. 462-480.
19. Odert, P., et al., *Escape and fractionation of volatiles and noble gases from Mars-sized planetary embryos and growing protoplanets.* Icarus, 2018. **307**: p. 327-346.
20. Krissansen-Totton, J., et al., *Oxygen false positives on habitable zone planets around sun-like stars.* AGU Advances, 2021. **2**(e2020AV000294).
21. Wordsworth, R., L. Schaefer, and R. Fischer, *Redox Evolution via Gravitational Differentiation on Low-mass Planets: Implications for Abiotic Oxygen, Water Loss, and Habitability.* The Astronomical Journal, 2018. **155**(5): p. 195.
22. Owen, J.E. and H.E. Schlichting, *Mapping out the parameter space for photoevaporation and core-powered mass-loss.* Monthly Notices of the Royal Astronomical Society, 2024. **528**(2): p. 1615-1629.
23. Birky, J., R. Barnes, and D.P. Fleming, *Improved Constraints for the XUV Luminosity Evolution of Trappist-1.* Research Notes of the AAS, 2021. **5**(5): p. 122.
24. Agol, E., et al., *Refining the transit-timing and photometric analysis of TRAPPIST-1: masses, radii, densities, dynamics, and ephemerides.* The planetary science journal, 2021. **2**(1): p. 1.
25. Schlichting, H.E. and E.D. Young, *Chemical Equilibrium between Cores, Mantles, and Atmospheres of Super-Earths and Sub-Neptunes and Implications for Their Compositions, Interiors, and Evolution.* The Planetary Science Journal, 2022. **3**(5): p. 127.
26. Hinkel, N.R., et al., *Stellar abundances in the solar neighborhood: the Hypatia Catalog.* The Astronomical Journal, 2014. **148**(3): p. 54.
27. Selsis, F., et al., *A cool runaway greenhouse without surface magma ocean.* Nature, 2023. **620**(7973): p. 287-291.

28. Krissansen-Totton, J. and J.J. Fortney, *Predictions for Observable Atmospheres of Trappist-1 Planets from a Fully Coupled Atmosphere–Interior Evolution Model.* The Astrophysical Journal, 2022. **933**(1): p. 115.
29. Costa, A., L. Caricchi, and N. Bagdassarov, *A model for the rheology of particle-bearing suspensions and partially molten rocks.* Geochemistry, Geophysics, Geosystems, 2009. **10**(3).
30. Salvador, A. and H. Samuel, *Convective outgassing efficiency in planetary magma oceans: insights from computational fluid dynamics.* Icarus, 2023. **390**: p. 115265.
31. Delrez, L., et al., *Two temperate super-Earths transiting a nearby late-type M dwarf.* Astronomy & Astrophysics, 2022. **667**: p. A59.
32. Anglada-Escudé, G., et al., *A terrestrial planet candidate in a temperate orbit around Proxima Centauri.* Nature, 2016. **536**(7617): p. 437-440.
33. Baraffe, I., et al., *New evolutionary models for pre-main sequence and main sequence low-mass stars down to the hydrogen-burning limit.* Astronomy & Astrophysics, 2015. **577**: p. A42.
